# Supplementary material for: Mechano-Transduction Boosts the Aging Effects in Human Erythrocytes Submitted to Mechanical Stimulation
Source: Int J Mol Sci. 2022 Sep 5;23(17):10180. doi: 10.3390/ijms231710180 (PMC9456273; doi:10.3390/ijms231710180)
Supplement: Supplementary file 1 [file ijms-23-10180-s001.zip › ijms-1861506-supplementary.pdf]

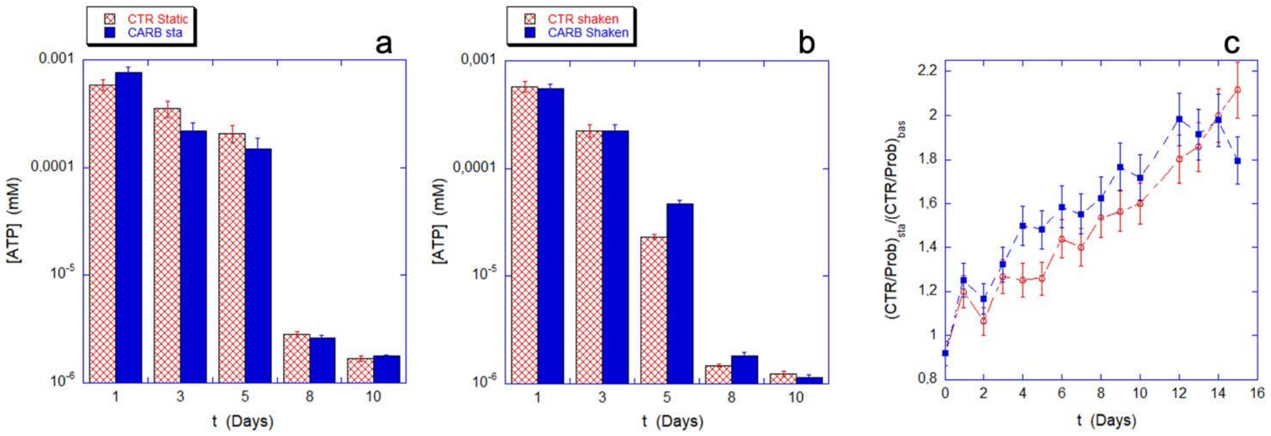

Figure S1: behavior of carbenoxolone

The figure shows two data cited, but not shown, in the text. They concern with the behavior of carbenoxolone, which has the same molecular target of the probenecid. It is interesting to note that its behavior is practically identical to that of probenecid both in terms of reduction of the ATP consumption (panel a,b) and in term of overall cell lysis compared to controls (c).
